# Proportion of Concentrate in the Diet of Early Lactation Dairy Cows Has Contrasting Effects on Circulating Leukocyte Global Transcriptomic Profiles, Health and Fertility According to Parity

**DOI:** 10.3390/ijms24010039

**Published:** 2022-12-20

**Authors:** Zhangrui Cheng, Danielle L. McLaughlin, Mark W. Little, Conrad Ferris, Mazdak Salavati, Klaus L. Ingvartsen, Mark A. Crowe, D. Claire Wathes, the GplusE Consortium

**Affiliations:** 1 Department of Pathobiology and Population Sciences, Royal Veterinary College, Hatfield AL9 7TA, UK; 2 Agri-Food and Biosciences Institute, Belfast BT9 5PX, UK; 3 The Roslin Institute, Royal (Dick) School of Veterinary Studies, Easter Bush Campus, The University of Edinburgh, Edinburgh EH25 9RG, UK; 4 Department of Animal and Veterinary Science, Aarhus University, 8000 Tjele, Denmark; 5 School of Veterinary Medicine, University College Dublin, Belfield, D04 V1W8 Dublin, Ireland

**Keywords:** postpartum immunosuppression, innate immunity, metabolism, leukocytes, lactation diets, transcriptome, reproduction, cows

## Abstract

The functionality of circulating leukocytes in dairy cows is suppressed after calving, with negative energy balance as a risk factor. Leukocyte transcriptomic profiles were compared separately in 44 multiparous (MP) and 18 primiparous (PP) Holstein–Friesian cows receiving diets differing in concentrate proportion to test whether immune dysfunction could be mitigated by appropriate nutrition. After calving, cows were offered either (1) low concentrate (LC); (2) medium concentrate (MC) or (3) high concentrate (HC) diets with proportions of concentrate to grass silage of 30%:70%, 50%:50% and 70%:30%, respectively. Cow phenotype data collected included circulating metabolites, milk yield and health and fertility records. RNA sequencing of circulating leukocytes at 14 days in milk was performed. The HC diet improved energy balance in both age groups. There were more differentially expressed genes in PP than MP cows (460 vs. 173, HC vs. LC comparison) with few overlaps. The MP cows on the LC diet showed upregulation of the complement and coagulation cascade and innate immune defence mechanisms against pathogens and had a trend of more cases of mastitis and poorer fertility. In contrast, the PP cows on the HC diet showed greater immune responses based on both gene expression and phenotypic data and longer interval of calving to conception. The leukocytes of MP and PP cows therefore responded differentially to the diets between age, nutrient supply and immunity affecting their health and subsequent fertility.

## 1. Introduction

Early postpartum, dairy cows are characterised by immunosuppression affecting innate and adaptive immunity, including both cell-mediated and humoral immunity. Circulating leukocytes are recruited into tissues such as the mammary gland and endometrium during inflammation. Previous studies have, however, shown a reduction in both leukocyte numbers and their functional capacity during the peripartum period [1,2]. Reported effects include impaired phagocytosis and oxidative burst activity [3,4,5], a decreased responsiveness of circulating T-cells to mitogenic agents and reduced production of immunoglobulin by B-cells [6,7]. The causes are multifactorial but appear linked to nutrient partitioning in favour of milk production at the start of lactation, compromising immune function [8,9,10].

Leukocytes require an adequate supply of glucose, various fatty acids and cholesterol or oxysterols for their maintenance and functionality [8,11,12]. The available nutrient supply is, however, prioritised to the mammary gland after calving, requiring approximately 25% more metabolisable energy and protein than is provided by feed intake [8,13,14]. In addition, parturition is characterised by inflammatory processes associated with changes in the secretion of various prostaglandins, steroids and cytokines and possible injuries during the delivery process itself [15,16,17]. The inflammatory responses to calving and metabolites released during tissue mobilisation have potent anorexic effects, further decreasing intakes and exacerbating the negative energy balance (NEB) [18]. When feed intake cannot meet the increasing energy demand, then cows enter a period of NEB, with some individuals becoming metabolically imbalanced [19,20,21].

NEB is associated with insulin resistance, reduced hepatic growth hormone (GH) receptor expression and lower hepatic IGF-1 synthesis [22,23]. The uncoupling of GH with insulin in postpartum cows is an adaptation which prioritises the glucose supply to tissues such as mammary epithelial cells, in which uptake is independent of insulin [8]. In this situation, the competition for energy supply between the mammary gland and immune system is unavoidable as they both rely on the same essential substrates and both are major consumers of energy. Kvidera et al. [24] demonstrated that activation of a full immune response in cows required 2.5 to 3.1 Kg of glucose per day. This is similar to the estimated requirement of 2.7 kg/d of glucose taken up by mammary epithelial cells for a milk production of 40 kg/d [8].

Tissue mobilisation is also associated with increased circulating concentrations of non-esterified fatty acids (NEFAs), beta-hydroxybutyrate (BHB), and decreased IGF-1, all of which contribute to immune dysfunction [5,9,10,20]. For example, our recent study illustrated that leukocyte cell-to-cell adhesion was inhibited when the NEFA concentration exceeded 750 μM [25]. Although tissue mobilisation is a normal mammalian adaptation to support lactation, many studies have shown that severe NEB has a major influence on the cow’s subsequent ability to conceive in a timely fashion [26]. This can be due to a variety of factors including delays in restoration of an appropriate uterine environment [2]; an extended period of anovulation [27], a poor intrafollicular environment [28] and impaired oocyte quality [29].

Dairy heifers generally calve at around 90% of their mature body weight [30]. Our previous study showed that primiparous (PP) cows had higher insulin, IGF-1 and leptin concentrations in their circulation, lower concentrations of blood metabolites (BHB, NEFAs and urea) and higher glucose concentration than multiparous (MP) cows for at least 7 weeks after calving [31,32]. This indicates that there is less uncoupling of the somatotrophic axis in early lactation in PP cows compared to the MP cows, associated with weaker prioritisation of nutrients to the mammary gland, so allowing continued growth. The gene expression profiles in circulating leukocytes also differ between PP and MP cows during early lactation [33].

Optimising nutritional management during the dry period can increase dry matter intake (DMI), reduce the incidence of periparturient disease and improve fertility [34]. There are general guidelines for how to reduce disease risk related to feeding and management [35]. There is, however, little information available on dietary formulations for the individual cow in metabolic imbalance during the postpartum period that can improve immune function, decrease disease incidence and improve subsequent reproductive performance. Formulating diets with a high nutrient intake potential is a practical approach [36]. In the present study, cows in early lactation were offered low (LC), medium (MC) or high concentrate (HC) diets based on the proportions of concentrate to grass silage, and effects on metabolic health and disease, and systemic immunity (by assessing global circulating leukocyte gene expression profiles) were determined. Data from PP and MP cows were analysed separately to avoid any confounding effect arising from parity. Our hypothesis was that the high concentrate diets would improve immune function and benefit fertility.

## 2. Results

### 2.1. Effect of Diets on Dry Matter Intakes, Milk Yield, Energy Balance and Blood Metabolites

Milk parameters, body weight (BW), DMI, body condition score (BCS), energy balance (EBAL) and blood metabolites for both MP and PP cows at around 14 days in milk (DIM) are summarised in Table 1. DMI for the MP cows were significantly different between the three dietary groups with the order of HC > MC > LC (*p* < 0.01–0.001). The milk yield in the MP cows receiving the HC diets was significantly higher than those receiving the LC diets (*p* < 0.05). The difference of energy corrected milk yield (ECM) between the LC and HC groups was significant (*p* < 0.05), with intermediate values in the MC group. The EBAL values in all three MP groups were negative but those in the MC and HC cows were significantly better (less negative) than in the LC cows (*p* < 0.01). The circulating concentrations of both glucose (*p* < 0.01) and IGF-1 (*p* < 0.001) in the HC cows were significantly higher than in the LC cows. The MP cows offered HC diets also produced less circulating urea (*p* < 0.001), BHB (*p* < 0.05) and NEFAs than those offered LC diets with the concentrations LC > MC > HC, although the differences of NEFA concentrations between the dietary groups were not statistically significant.

In the PP cows, those receiving the MC or HC diet had a higher DMI than those receiving the LC diet (*p* < 0.01). Milk yields were not significantly different. The EBAL was positive in cows offered MC or HC diets whereas for those offered the LC diet it was negative. The differences of EBAL between HC and LC PP cows were significant (*p* < 0.05). Of the metabolites measured, only urea concentrations differed between the groups, being lower in the HC cows (LC > MC > HC, *p* < 0.001 for LC vs. HC or MC).

### 2.2. Effect of Diets on Inflammatory Parameters

The effects of diet on inflammatory parameters measured in the uterus and mammary gland are presented in Table 2. In the PP cows, the ratio of polymorphonuclear leukocytes to uterine epithelial cells (PMNs:UECs) collected from the uterus using a cytobrush was significantly higher for the cows on the HC compared with the LC diet (*p* < 0.05). In the MP cows, the trend was in the same direction, with LC < MC < HC, but did not achieve statistical significance. In milk, the MP cows on the LC diet had a significantly higher SCC than those on either the MC or HC diets (*p* < 0.05), whereas in PP cows the non-significant trend was in the opposite direction. Milk N-acetyl-β-d-glucosaminidase (NAGase) and lactate dehydrogenase (LDH) concentrations, milk enzymes indicative of mastitis, followed the same general trends as the SCC values but no differences were significant. These results were supported by the health records. The MP cows on the HC diet did not experience any cases of mastitis (0/15) whereas 4/14 (28.6%) of cows on the LC diet were diagnosed with clinical mastitis within 16 days of calving. The trend was again in the opposite direction for the PP cows with one of six (16.7%) on the LC diet with clinical mastitis compared with one case of clinical and two of subclinical mastitis on the HC diet (50%).

### 2.3. Fertility Data

Details of the fertility data are given in Table 3. For the MP cows, those on the LC diet took slightly longer to conceive and required more services per conception than the HC cows (2.4 ± 0.42 vs. 1.6 ± 0.23), although more of the HC cows were either not served (a management decision) or failed to conceive at all (60.0% vs. 71.4%). None of these differences were, however, significant. For the PP cows, the reverse was true, with the LC cows having the shortest interval to conception by 29 days (*p* < 0.05), with five of the six animals conceiving to their first service. In contrast, half of the HC group were either not served (n = 2) or failed to conceive (n = 1), with the three which did conceive requiring 2.0 ± 0.41 service per conception. This resulted in the HC group having a significantly higher “in calf by” (ICB) score (*p* < 0.05).

### 2.4. Leukocyte Transcriptomic Profiles in Cows Offered Different Proportions of Concentrate

**Figure 1:**
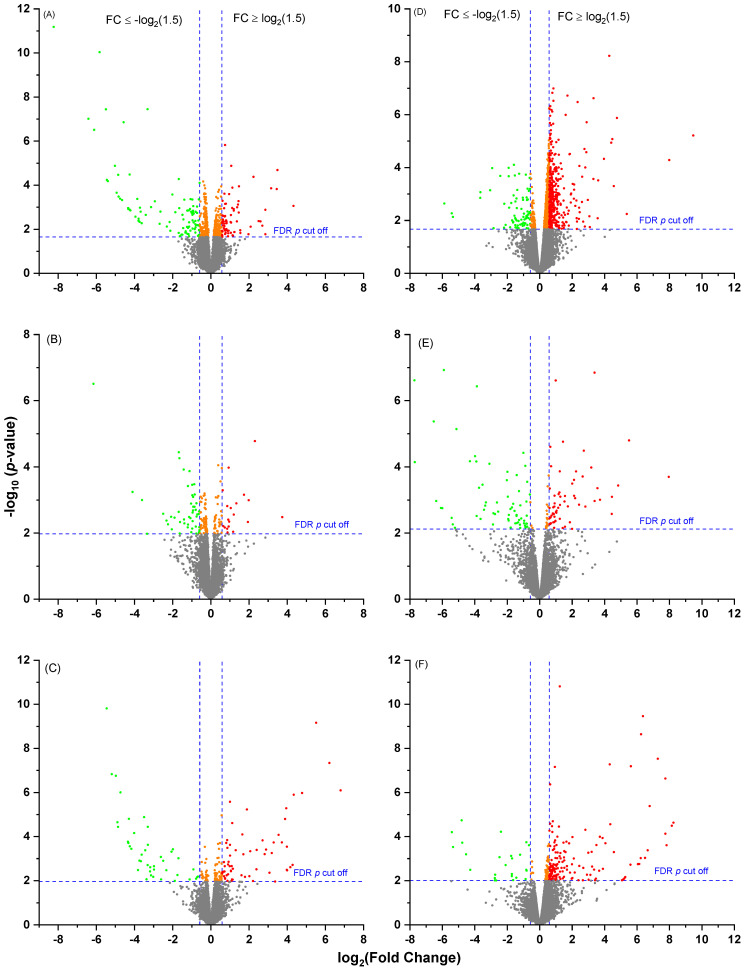
Volcano plots showing the expression profiles in both the MP and PP cows receiving three different diets. (**A**) MP cows HC vs. LC, (**B**) MP cows HC vs. MC, (**C**) MP cows MC vs. LC, (**D**) PP HC vs. LC, (**E**) PP HC vs. MC and (**F**) PP MC vs. LC. HC: high concentrate (n = 6 in PP cows and n = 14 in MP cows), MC: medium concentrate (n = 5 in PP cows and n = 15 in MP cows) and LC: low concentrate (n = 6 in PP cows and n = 14 in MP cows). The fold changes were log2-transformed. The green dots indicate the downregulated genes with *p* (BH) < 0.05 and fold changes ≤ −1.5 and red dots indicate upregulated genes with *p* (BH) < 0.05 and fold changes ≥ 1.5. The orange dots indicate the genes with *p* (BH) < 0.05 but absolute fold changes < 1.5.

**Figure 2:**
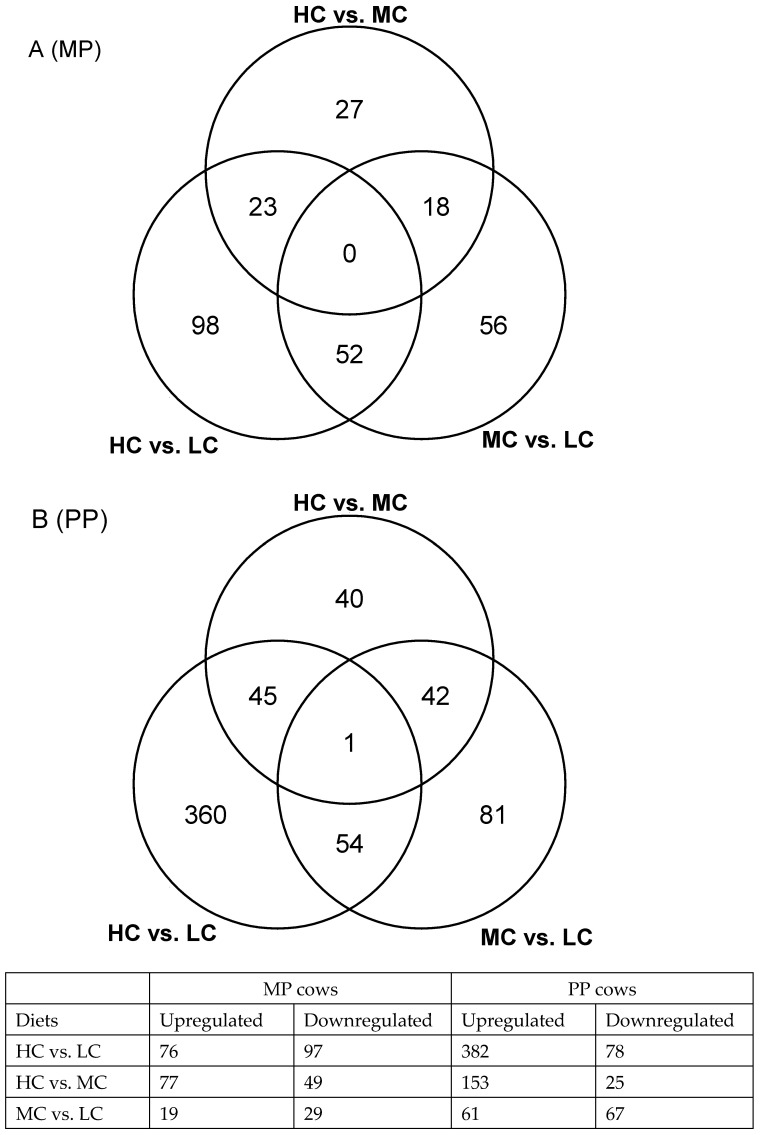
Venn diagrams showing the differentially expressed genes by the circulating leukocytes between the three dietary groups in (**A**) multiparous (MP) cows and (**B**) primiparous (PP) cows. HC: high concentrate (n = 6 in PP cows and n = 14 in MP cows), MC: medium concentrate (n = 5 in PP cows and n = 15 in MP cows) and LC: low concentrate (n = 6 in PP cows and n = 14 in MP cows).

**Figure 3:**
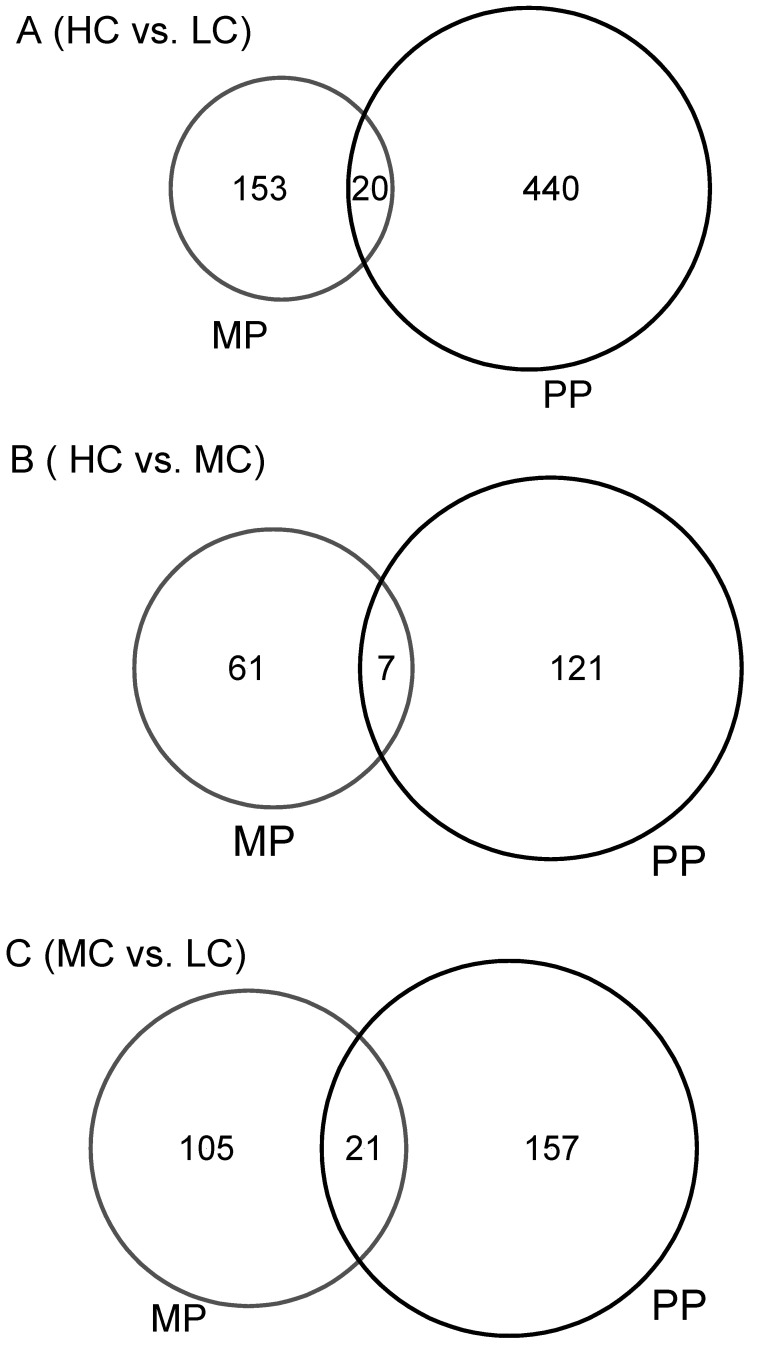
Venn diagrams showing the differentially expressed genes by circulating leukocytes between the PP and MP cows in the comparisons of: (**A**) high concentrate (HC) with low concentrate (LC); (**B**) HC with medium concentrate (MC), and (**C**) MC with LC. HC: n = 6 in PP cows and n = 14 in MP cows, MC: n = 5 in PP cows and n = 15 in MP cows and LC: n = 6 in PP cows and n = 14 in MP cows.

The reference bovine genome of ARS-UCD 1.2 provided by RefSeq (https://www.ncbi.nlm.nih.gov/assembly, accessed on 1 May 2022) contains 35,158 genes, of which 19,001 leukocyte genes were quantifiable when the sequencing reads in the FASTQ files were mapped to it. Volcano plots showing the expression profiles in both the MP and PP cows receiving the three different diets are presented in Figure 1. In the MP cows, there were 173 differentially expressed genes (DEGs) in the comparison between HC and LC, 126 between MC and LC and 68 for HC vs. MC (Supplementary File S1A–C). Venn diagram illustrated that no gene (common DEG) was significant in all three dietary comparisons (Figure 2A). In the PP cows, there were 460 DEGs in the comparison of HC vs. LC, 178 between MC vs. LC and 128 between HC vs. MC (Supplementary File S2A–C). Just one common gene (*DCN*, fold change (FC) = −7.3) was significant in all three dietary comparisons (Figure 2B). This encodes decorin, a protein which plays a role in collagen fibril assembly. Overall it was noticeable that a greater number of DEGs were found in the dietary comparisons of leukocyte gene expression in the PP than the MP cows despite the smaller group sizes, with the majority of these genes being upregulated on the HC diet. Furthermore, there was little overlap in the genes identified between the different age groups, as illustrated in the Venn diagrams in Figure 3. This suggests that the diets had different effects on leukocyte transcriptome in PP and MP cows. In both age groups, the greatest differences were found between the cows offered the LC vs. HC diets, so we focused on this comparison in the subsequent analyses.

### 2.5. Comparison of Leukocyte Gene Expression Profiles between Multiparous Cows Receiving High or Low Concentrate Diets

The top 20 upregulated and downregulated DEGs ranked by the *p* values (BH adjusted) in the MP cows fed a HC compared with those fed a LC diet are given in Supplementary File S1D,E. Among the upregulated DEGs, most were involved in the GO functions of immune system process, metabolism and response to stimulus, with many encoding proteins having multiple roles. For example, *ALAS2* (encoding 5′-aminolevulinate synthase 2) has a role in metabolic process, response to stimulus and developmental process. *COL1A1* (collagen type I alpha 1 chain) and *DAB2* are involved in metabolism, response to stimulus, multicellular organism, locomotion and development. The top 20 downregulated genes illustrate a clear theme of changes in immunity with 14 associated DEGs. Of these, *DMTB1*, *FGA*, *FGB* and *TF* encode antimicrobial peptides, *ALB* and *TF* encode negative acute phase proteins (APP), and *FGA* and FGG encode positive APPs. Eight genes play roles in metabolism and 10 are involved in response to stimulus.

**Figure 4:**
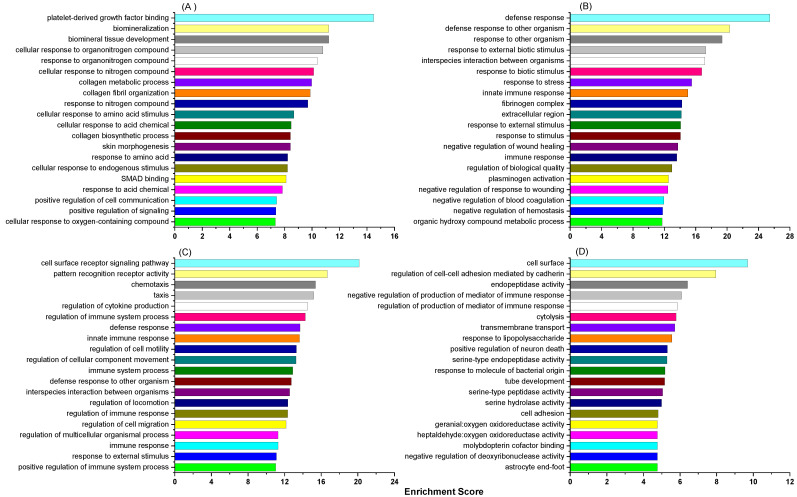
Top 20 GO functions associated with the DEGs derived from HC vs. LC comparisons. (**A**) MP upregulated DEGs, (**B**) MP downregulated DEGs, (**C**) PP upregulated DEG and (**D**) PP downregulated DEGs.

The leukocyte DEGs derived from the comparison of the HC with the LC group were next subjected to GO enrichment analysis. The 76 upregulated DEGs were significantly enriched with 208 functions, of which the top 20 based on the enrichment scores are shown in Figure 4A. These were associated with various aspects of cellular function, with platelet-derived growth factor binding on top. Many functions were related to protein and amino acid metabolism (involving *APLP1*, *COL1A1*, *COL1A2*, *COL3A1*, *HTR1B* and *P2RY12* in most of them), biomineralisation and collagen processing (involving *COL1A1*, *COL1A2*, *SPP1* and *TUFT1*), and cell communication. In contrast, the top functions of the 97 downregulated DEGs had a clear theme of various immune defence processes (Figure 4B) which were predominantly associated with 20 DEGs. Of these, *FGA* (a positive APP) was downregulated by 85 fold in the HC vs. LC comparison. A GO browser tool summarised the biological functions of both up and down regulated DEGs into eight significant categories (Table 4). Except for biomineralisation, all the other biological functions, especially those associated with immune defence, were predominantly associated with the downregulated DEGs.

The combined up and downregulated DEGs (n = 173) were significantly enriched with 23 KEGG pathways which were mainly related to various immune and metabolic processes (Table 5). Complement and coagulation cascades were on the top with eight downregulated DEGs (*CFB*, *FGA*, *FGB*, *FGG*, *KNG1*, *PROC*, *SERPINA1* and *VTN*). The inflammatory pathway of NOD-like signalling was associated with six DEGs (*IFNB1*, *MAPK10*, *OAS1X*, *OAS1Y*, *OAS1Z* and *OAS2*), of which all but *MAPK10* were downregulated. The pathway of protein digestion and absorption contained six DEGs, of which three (*COL1A1*, *COL1A2* and *COL3A1*) were upregulated and three downregulated (*CELA2A*, *COL5A3* and *ELN*). Five downregulated DEGs were involved in the peroxisome proliferator-activated receptors (PPARs) signalling pathway (*APOA2*, *APOC3*, *FABP1*, *HMGCS2* and *PCK1*). The pathways of extracellular matrix–receptor interaction, focal adhesion and sphingolipid signalling are all involved in the maintenance of cell and tissue structure.

### 2.6. Comparison of Leukocyte Gene Expression Patterns between Primiparous Cows Receiving High or Low Concentrate Diets

The top 20 circulating leukocyte DEGs in the PP cows fed the HC compared with the LC diets are listed in Supplementary File S2D,E. Immune and metabolic processes predominated the biological functions of the top upregulated DEGs, containing 11 and 12 DEGs respectively. Among these, *ACSL6*, *MMP9* and *SLC11A1* are involved in leukocyte proliferation, and *ACSL6*, *ADGRG3*, *COL1A2*, *DUSP1*, *HCK*, *MMP9* and *PADI4* in developmental process. *SLC40A1* is a major iron transporter that plays a key role in balancing cellular and systemic iron levels. Some of these DEGs encode proteins with multiple functions. For example, *MMP9*, *DUSP1*, *SLC11A1* and *COL1A2* are associated with most of the above GO functions. The biological functions of the top downregulated DEGs were more diverse. There were eight DEGs involved in immune system process, five playing roles in metabolism, and two associated with both leukocyte proliferation and developmental process (*EPCAM* and *FCRL3*). Again, some DEGs, for example *CD96* and *FCRL3*, encode proteins with multiple roles.

In the PP cows, 382 upregulated DEGs derived from the HC vs. LC comparison were significantly associated with 690 GO functions, with the top 20 presented in Figure 4C. All these involved 116 DEGs (Supplementary File S2F) with various immune activities. The top function was cell surface receptor signalling pathway. This was associated with 43 DEGs which included genes encoding many receptors for immune ligands, such as *CXCR1*, *CXCR2*, *IL17RD* and *IL1RAP*. This function had six significant sub-functions including the lipopolysaccharide (LPS)-mediated signalling pathway (*PTAFR*, *TLR4* and *SCARB1*) and immune response-regulated cell surface receptor signalling pathway. The top sub-function within regulation of multicellular organismal process was regulation of cytokine production, with 24 upregulated DEGs, such as *MARK13*, *LTF* and *TLR4*.

There were fewer downregulated DEGs (only 78) which were associated with 298 significant GO functions, with lower enrichment scores and more diverse biological processes. Figure 4D shows the top 20 functions. A number of these were associated with aspects of immunity, such as cell adhesion, regulation of production of mediator of immune response, response to LPS, response to molecule of bacterial origin. Others were related to maintenance of homeostasis, such as cell surface, cytolysis, transmembrane transport, serine-type endopeptidase.

Summarisation of the significant biological functions associated with both up and downregulated DEGs generated seven categories (Table 6), with immune system process on top and most of the other functions also associated with immune defence. For example, locomotion was enriched by the DEGs with chemotactic and immune properties and interspecies interaction between organisms, which involves killing invaded pathogens. Both cellular process and biological regulation were associated with large numbers of DEGs. Cellular process (n = 239 DEGs) included many sub-functions associated with cell adhesion, cell killing, immune cell activation and cell population proliferation. Biological regulation (125 DEGs) also involved many immune activities, such as regulation of immune system process, locomotion and response to stimulus. All of these functions contained predominantly upregulated DEGs on the HC diet.

The combined up and downregulated DEGs (460) in the PP cows were enriched with 35 significant KEGG pathways (Table 7). Many were associated with metabolic processes involving amino acids (valine, leucine, isoleucine, arginine and glutathione), proteins, lipids (arachidonic acid, glycerolipid and cholesterol), vitamin B6 and some hormones (aldosterone, cortisol, thyroid and growth hormone). These metabolic pathways were predominantly enriched with the DEGs upregulated on the HC diet. For example, biosynthesis of amino acids was associated with five upregulated DEGs (*ARG2*, *ASS1*, *GPT2*, *SDS* and *SDSL*), the pathway of growth hormone synthesis, secretion and action was enriched with six upregulated (*ADCY6*, *CREB3L2*, *CREB5*, *FOS*, *MAPK13* and *SOCS3*) and one downregulated (*BCAR1*) DEGs, and arachidonic acid metabolism contained five upregulated DEGs (*CYP2J2*, *GGT5*, *GPX3*, *PLB1* and *TBXAS1*). Several pathways associated with immune/inflammatory process were also significantly enriched, again mainly with upregulated DEGs. These included chemokine, MAPK and TNF signalling pathways and complement and coagulation cascades. For example, the chemokine signalling pathway contained eight upregulated (*ADCY6*, *CCL16*, *CCR1*, *CXCL13*, *CXCR1*, *CXCR2*, *GNG7* and *HCK*) and two downregulated DEGs (*BCAR1* and *CCR5*), the TNF signalling pathway contained six upregulated DEGs (*CREB3L2*, *CREB5*, *FOS*, *MAPK13*, *MMP9* and *SOCS3*), and the MAPK signalling pathways contained 12 upregulated DEGs, including *IL1A*, *MAP3K6* and *MAPK13*.

## 3. Discussion

Postpartum dairy cows have homeorhetic mechanisms to control nutrient partitioning between lactation and other important life functions, such as immunity and growth [8]. Tissue mobilisation is a normal mammalian adaptation to support lactation, but some cows become metabolically imbalanced [35,37]. Such animals experience excessive adipose tissue mobilisation, insulin resistance and systemic inflammation which contribute to the impaired immunity often observed at this time [38]. This is associated with inflammatory mediators such as TNF and IL6 [39,40]. On day 14 after calving, when the circulating leukocyte samples in this study were collected, the cells had therefore been exposed to a period of inflammatory stimulation. We have demonstrated using next generation sequencing and bioinformatics analysis that the diets with different proportion of concentrate produced different effects on the circulating leukocyte transcriptome in PP and MP cows in early lactation. This was associated with differences in their health and fertility. This information has added new findings to our previous report of the dietary effects on milk production and immunity [32].

### 3.1. Comparison of the Effects of the High and Low Concentrate Diets in the Multiparous Cows

The MP cows on the HC diets had a higher DMI than MC and LC cows and this was associated with higher circulating concentrations of both glucose and IGF-1. This led to the HC cows being in less severe NEB and producing more milk. The HC diet was formulated to meet the energy and protein requirements for both lactation and maintenance of body homeostasis and the metabolite measurements confirmed that their metabolic status was indeed better than for the LC cows, with a lesser requirement for tissue mobilisation to meet energy demands. This should help to speed up the resolution of postpartum inflammatory processes, as many inflammatory diseases in early lactation are associated or caused by metabolic disorders [38] and we showed previously that uterine inflammation was resolved more quickly in cows with a better energy balance status [2]. In the present study, there was no difference in the ratio of PMNs to epithelial cells in the uterine lumen according to diet in the MP cows. This index is often taken as an indicator of cytological endometritis [41]. Differences according to diet were, however, observed in the mammary gland as the LC cows had a higher SCC and this was associated with a greater proportion of them experiencing clinical mastitis during the first 16 DIM (28.6% vs. 0% in the LC vs. HC groups).

Most of the DEGs identified in the analysis of the leukocyte transcriptome were involved in immune and/or metabolic processes, as expected in an immune cell population. The most significant KEGG pathway was the complement and coagulation cascade, which included six of the most highly downregulated genes in the HC cows. Of these, *CFB* encodes complement factor B, a component of the alternative pathway of complement activation. *FGA*, *FGB* and *FGG* encode three subunits of the coagulation factor fibrinogen, a key component of the blood clot that is also important in binding bacteria to platelets [42]. The high molecular weight form of kininogen, encoded by *KNG1*, is also essential for blood coagulation and kininogen releases bradykinin, a peptide with various functions including antibacterial and antifungal activity. Vitronectin, encoded by *VTN*, is also involved in regulation of the coagulation pathway and wound healing, while its heparin-binding domain provides anti-microbial properties. *PROC* encodes a plasma glycoprotein whose activated form contains a serine protease domain that functions in degradation of the activated forms of coagulation factors V and VIII, while *SERPINA1* encodes a serine protease inhibitor whose targets include plasmin, thrombin, and plasminogen activator. Upregulation of the complement and coagulation cascade has previously been shown in mammary tissue as an early host response to *E. coli* or *Staph. aureus* infection [43]. As this pathway was more highly expressed in the cows on the LC diet, this supports evidence of a greater incidence of mastitis in these animals.

Another top function summarised by GO browser was the pathogen killing process (interspecies interaction between organisms), which was associated with 14 downregulated DEGs in the HC cows (Table 4). This included a number of genes involved in antiviral activity (*IFI6*, *IFNB1*, *ISG15*, *MX2*, *OAS1Y*, *OAS1Z*, *OAS2* and *RSAD2*) that were expressed at lower levels. Of these, *IFNB1* encodes interferon beta 1 while the others are all interferon stimulated genes [44,45]. They are all also part of the NOD-like receptor (NLR) signalling pathway. NLRs are cytosolic pattern recognition receptors which are activated by various non-self-components including bacterial peptidoglycan, potentially initiating NF-kappa B-/AP-1-dependent expression of pro-inflammatory cytokines, expression of type I interferons, autophagy and inflammation [46]. Our recent study demonstrated that this pathway was upregulated in leukocytes from *E. coli*-infected cows [47]. Another gene identified was *MPO*, encoding myeloperoxidase, an enzyme stored in azurophilic granules of PMNs and macrophages. It is released into extracellular fluid during inflammatory processes and has been used as a marker of inflammation and oxidative stress [48]. *IL1R2* encodes a member of the interleukin 1 receptor family and was one of the most significantly downregulated DEG identified in the MP cows. This can bind IL1A, IL1B, and interleukin 1 receptor, type I (IL1R1/IL1RA) but acts as a decoy receptor to inhibit ligand activity.

The gene with the most significant differential expression between MP cows on the different diets was *FCER1A*, with lower expression in the HC cows. This encodes a subunit of the IgE receptor, which is the initiator of allergic responses that might have evolved to promote host defence against parasites through release of mediators such as histamine [49]. Interestingly, *FCER1A* was one of only 18 genes whose expression level was identified as discriminating between fertility of beef heifers [50]. Its precise role in cattle remains undetermined but in a vaccine trial of calves experimentally infected with the economically important parasitic nematode *Ostertagia ostertagi*, its expression levels in blood correlated positively with mast cell counts and negatively with the number of worms [51].

Three other upregulated genes in the HC cows with some immune function were *ALAS2*, *GZMB* and *LIF*. *ALAS2* encodes 5′-aminolevulinate synthase 2, an erythroid-specific mitochondrially located enzyme that catalyses the first and rate-limiting step in the heme biosynthetic pathway. Heme is an essential cofactor in a variety of key processes including oxygen transport, while mutations in this gene have been linked to a variety of human diseases including anaemia [52]. *GZMB* encodes a preproprotein that is secreted by natural killer cells and cytotoxic T lymphocytes and is processed to generate an active protease that induces target cell apoptosis and also processes cytokines and degrades extracellular matrix proteins [53]. The cytokine leukemia inhibitory factor, encoded by *LIF*, was initially identified through involvement in macrophage differentiation but it has also been shown to play an important role in embryo development and establishment of pregnancy in a variety of species including cattle [54].

The DEGs derived from the HC vs. LC comparison in the MP cows were also enriched with several significant pathways associated with glucose, protein and fatty acid metabolism. These included four downregulated DEGs involved in PPAR signalling pathways (*APOA2*, *APOC3*, *FABP1* and *PCK1*). In this study, three major PPAR isoforms (A, D, G) were detected in the leukocyte population. Although expression of the PPARs themselves was not influenced by diet, this pathway can influence expression of genes involved in glucose and lipid metabolism, adipocyte differentiation and inflammatory responses [55,56] and contributes to the metabolic adaptation to a limited nutrient supply by inducing genes involved in β-oxidation [57]. The retinol metabolic pathway was also downregulated in the HC cows, with lower expression of *RBP4* and *TTR* (encoding retinol binding protein and transthyretin, respectively), both of which act as retinol transporters in the blood. Interaction of retinol with the PPAR signalling pathway influences transcription of many other downstream genes [58]. *APOA2* and *APOC3* together with *APOH* are also part of the cholesterol metabolic pathway. Cholesterol is a major component of the plasma membrane and influences both its organisation and function [59,60]. Recent studies have underlined an emerging role for cholesterol as an important modulator of innate and adaptive immune activity [61].

The pathway of glycolysis/gluconeogenesis was associated with three downregulated DEGs in the HC fed cows (*ADH1C*, *ALDOB* and *PCK1*). Of these, *PCK1* encodes phosphoenolpyruvate carboxykinase 1 which acts as the main control point for the regulation of gluconeogenesis. This enzyme, together with GTP, catalyses the formation of phosphoenolpyruvate from oxaloacetate, with the release of carbon dioxide and GDP. Activation of immune/inflammatory pathways promotes the transcription of gluconeogenic genes via toll-like receptor 4 (TLR4) [62]. This can cause immune cells to switch their glucose metabolism from oxidative phosphorylation towards glycolysis to produce both energy and nutrients required for proliferation and immune molecule production [12,63]. This leads to an increased demand for glucose which competes against the requirement by lactate production [64].

In summary, the gene expression differences between MP cows on the HC and LC diets provide evidence of greater upregulated immune activity and inflammation in the LC cows. This was accompanied by a higher incidence of mastitis. In contrast, genes encoding some proteins likely to benefit health and fertility such as *ALAS2* and *LIF* were upregulated in the HC cows. This supports the metabolic indices measured showing that there was an improved EBAL status in the MP cows on the HC diets, which probably helped to protect them against the development of infectious or metabolic disease.

### 3.2. Comparison of the Effects of the High and Low Concentrate Diets in the Primiparous Cows

In similarity to the MP cows, the PP cows on the HC diet also had higher DMI, by about 4 kg/d, but milk yield was not increased significantly. There were no significant differences in circulating concentrations of glucose, NEFA, BHB or IGF-1, with only urea being higher on the LC diet. In contrast, there were more significant differences in leukocyte gene expression between dietary groups than in the MP cows, with 460 vs. 173 DEGs identified in the HC vs. LC comparisons, of which 83% were upregulated in PP cows on the HC diet. Only a small proportion of the DEGs overlapped between the two age groups (Figure 3), suggesting that the changes in leukocyte function to the early lactation diet were more sensitive in PP cows than in MP cows.

We had expected that the HC diet would benefit the PP cows by increasing their DMI and fully meeting the calculated dietary undegradable and metabolisable protein requirements. EBAL was indeed improved on the HC diet but the gene expression analysis showed a much greater upregulation of genes involved in immune defense. The HC fed cows also had a higher ratio of PMNs:UEC in their uteri together with a numerically greater SCC and more cases of mastitis. This suggests that they were actually more, rather than less, prone to disease. We previously found that neutrophils collected from the PP cows in this study in the first three weeks of lactation had significantly higher phagocytic index and oxidative burst index in comparison with the MP cows, but the 2-way effects of diet and parity on these measurements were not reported [32].

The immune system processes identified in the HC vs. LC comparison included upregulation of cell surface receptor and pattern recognition signalling pathways, chemotaxis, cytokine production and leukocyte migration in the HC fed cows. Identified DEGs were associated with a variety of immune defence mechanisms, including a variety of antimicrobial peptides (AMP) (*CATHL6*, *CXCL13*, *DEFB1*, *LTF*, *PGLYRP1*, *PGLYRP4*, *SA100A8*, *SA100A9*, *S100A1* and *SLC11A1*). These can not only kill the invaded organisms directly but also assist by modulating other immune and antimicrobial processes [65,66,67]. Upregulation of antimicrobial peptides in the leukocyte transcriptomic profiles has previously been demonstrated in cows with clinical mastitis [47,68] and metritis/endometritis [69,70].

The leukocyte adhesion function was associated with 16 upregulated and five downregulated DEGs in the HC fed animals. Adhesion of leukocytes to capillary walls is a vital first step in enabling them to transmigrate from the blood and traffic towards sites of tissue damage, infection and inflammation [71]. Both cell–cell and cell–matrix interactions influence leukocyte phenotype, and dysregulation of adhesion pathways can lead to persistent leukocyte activation with unresolved inflammation [72]. Of the upregulated DEG identified, *ADAM8*, *ANGPTL3*, *CD24*, *ICAM3* and *THY1* are all involved in extravasation [73,74] while *FN1*, *NRP1*, *TNFAIP6* and *VCAN* have potential roles in leukocyte cell trafficking and function in inflamed tissues [72,75]. The genes encoding the chemokine receptors *CXCR1* and *CXCR2* were also upregulated in the HC fed cows; these are both important in stimulating chemotaxis of PMN toward sites of infection as well as activating biochemical processes that kill invading bacteria [76].

Of the downregulated genes associated with adhesion in the HC fed cows, *BCAR1* encodes a multifunctional protein known as cas with involvement in cell motility, apoptosis and cell cycle control [77]. Polymorphisms in *BCAR1* have previously been associated with SCC and mastitis resistance [78]. *ADGRG1* (also known as *GPR56*) encodes a G protein-coupled receptor that binds collagen 3 and transglutaminase 2, both components of tissue stroma. ADGRG1 was shown to play a role in human natural killer (NK) cells, in which it inhibits their cytotoxicity [79]. Its own expression is downregulated following cytokine-induced activation, which would accord with the results shown here. CD96 protein also acts as an inhibitory checkpoint receptor on NK cells [80]. Previous studies on changes in the leukocyte transcriptome during the transition period have also found changes in gene expression relating to transendothelial migration although with differing conclusions, reporting either activation after calving [81] or inhibition [82], respectively.

Our analysis also identified a number of differences in amino acid metabolism signalling pathways in PP cows on the HC diet compared with those on the LC diet, associated with enzymes encoded by five upregulated DEGs (*ARG2*, *ASS1*, *GPT2*, *SDS* and *SDSL*). Of these, *ASS1* encodes arginine succinate synthase 1, which catalyses the penultimate step of the arginine biosynthetic pathway, while *ARG2* encodes arginase, catalysing the hydrolysis of arginine to ornithine and urea. L-arginine can also be converted to nitric oxide, a signalling molecule that plays a key role in the pathogenesis of inflammation [83]. Glutamic-pyruvic transaminase 2 (*GPT2*) is a mitochondrial enzyme that catalyses the reversible transamination between alanine and 2-oxoglutarate to generate pyruvate and glutamate. This gene is upregulated under conditions of metabolic stress and plays roles in driving gluconeogenesis from amino acid metabolism [84]. Serine dehydratase (*SDS*) encodes an enzyme that converts L-serine to pyruvate and ammonia, while serine dehydratase like (*SDSL*) is predicted to be involved in the pathway of isoleucine biosynthesis from threonine.

The pathway of cholesterol metabolism was associated with four upregulated DEGs on the HC diet (*ANGPTL3*, *LRP1*, *SCARB1* and *SORT1*). This might lead to accumulation of cholesterol in the leukocytes of the HC fed cows and promote inflammation, including augmentation of TLR signalling, inflammasome activation, and greater production of monocytes and neutrophils in the bone marrow and spleen [85]. The pathway of arachidonic acid metabolism with five upregulated DEGs (as listed previously) leads to the production of cascades of pro- and anti-inflammatory products, such as prostaglandins and leukotrienes [86]. The glycerolipid metabolism pathway was associated with four upregulated DEGs (*DGAT2*, *DGKG*, *GK* and *GPAT3*) which encode for lipogenic genes involved in the synthesis of triacylglycerol. Its accumulation may induce leukocyte activation and inflammation [87,88].

All of these findings support the conclusion that the HC diet upregulated leukocyte metabolic pathways in the PP cows in a way that increased their immune/inflammatory responses. Previous work in cows has focused mainly on the dry period and has shown that overfeeding and high BCS at this time promotes subsequent lipid mobilisation and an increased inflammatory response during the peripartum period [34]. In the present study, the uterine PMN:UEC ratio and the trend towards a higher SCC support the suggestion that there was increased leukocyte migration to the uterus and mammary gland in the PP cows receiving the HC diet. While this is an essential component of immune defense, overactivation may contribute to hyper-inflammation. Further study with a larger sample size is required to confirm these findings.

### 3.3. Similarities in Response to Diet by Multiparous and Primiparous Cows

In terms of metabolic changes, both the MP and PP cows had higher circulating concentrations of urea when on the LC diet. Blood urea in both late pregnancy and early lactation may rise following mobilisation of amino acids stored in skeletal muscle [89] or when dietary protein supply exceeds energy availability or protein needed [90], so these situations could have applied here. Elevated levels of urea have been associated with reduced fertility, but only at >4.5 mmol/L, higher than the concentrations that were reached in the LC fed animals in the present study [91]. Regarding the leukocyte gene transcription data, it is interesting to note that the DEGs derived from the HC vs. LC comparison in both age groups were enriched with the pathway of protein digestion and absorption, in which the genes encoding various isoforms of collagen play important roles. Among them, there were three upregulated collagen genes (*COL1A1*, *COL1A2* and *COL3A1*) in both MP and PP cows, and one downregulated (*COL5A3*) collagen gene in the MP cows. Their expression values in the samples were small, but the differences were significant. Part of these RNA might come from fibrocytes, a cell population which comprises just 0.1–0.5% of non erythrocytic cells in peripheral blood [92,93]. Collagen is a long-established immune enhancer involved in many immune/inflammatory processes [93,94]. Its upregulated production in circulating blood by the HC diet may therefore affect leukocyte function.

### 3.4. Implications for Fertility

Many previous studies have reported that the interlinked metabolic and immune status and disease incidence of cows in early lactation has a major influence on their subsequent fertility (e.g., [38,95]). Our own work showed that MP cows with a low IGF-1 concentration at 14 DIM were less likely to conceive at all [37]. More recently, we reported that 63% of cows with low IGF-1 at this time experienced more than one health problem during their first 35 DIM in comparison with only 26% of high IGF-1 cows. This included more animals with uterine infections and clinical mastitis [10]. Foley et al. [69] differentiated between healthy cows, which were able to restore homeostasis within 3 weeks after calving, and others which experienced a more severe and prolonged inflammatory response which then went on to develop clinical endometritis. An association with peripheral blood was demonstrated by Galvão et al. [96] who showed that neutrophils from cows in worse EBAL had a lower glycogen content at seven DIM and these animals experienced more uterine disease, potentially associated with the decreased availability of oxidative fuels for immune responses. Clinical mastitis in early lactation is also associated with increased rates of pre-implantation embryo loss, with some indication that a low BCS further increased the risk [97]. A number of mechanisms associated with cytokine and prostaglandin production and other inflammatory mediators have been suggested which could affect the ovary and/or cause an unfavourable uterine environment [98,99].

The fertility data obtained from the cows in our study supported the findings relating to their immune status and health in early lactation. The MP cows on the LC diet required numerically more S/C in accord with their poorer EBAL together with evidence from analysis of the leucocyte transcriptome of more active immune defence in progress, as supported by their higher incidence of mastitis. In contrast, the PP cows on the HC diet took significantly longer to conceive and fewer became pregnant in comparison with the LC fed cows. They had more evidence of inflammation in early pregnancy even though their calculated EBAL was better. At present, the underlying mechanisms causing their poor health status and fertility is uncertain although we have also found evidence based on global gene expression of increased hepatic inflammation and fibrosis (Cheng, Little, Ferris, Takeda, Ingvartsen, Crowe and Wathes, unpublished observations).

### 3.5. Limitations of the Study

There were fewer PP cows per group available, and this reduced the statistical power of the analyses relating to the phenotypes. Further study with larger sample size is therefore required to confirm the dietary effects on reproduction and disease. In the present study, we extracted all RNA from whole peripheral blood using Tempus tubes and its isolation system. This offers ease of collection for a large scale on farm study but does not separate the cell types, which will include T and B lymphocytes, natural killer cells, platelets, monocytes, granulocytes (neutrophils, eosinophils and, basophils) and fibrocytes. The gene expression data presented have therefore been influenced by possible treatment effects on the relative proportions of particular cell types in addition to their individual transcriptional changes. These results are also based on gene transcription levels, and do not include information on their post-translational processing, which will also influence how much functional protein is produced.

## 4. Materials and Methods

### 4.1. Animals and Diets

All procedures were carried out under the Animals (Scientific Procedures) Act 1986 and covered by Home Office Project Licence number PPL2754 and a Certificate of Designation for the Establishment. The work was also approved by the Ethics and Welfare Committee of the Agri-Food and Biosciences Institute (AFBI, Belfast, Northern Ireland, UK). Sixty-two Holstein–Friesian dairy cows were recruited from the AFBI herd. Among them, 18 were PP (lactation 1) and 44 MP with lactation numbers 2–7 (3.5 ± 1.28) and all cows were healthy as examined by the veterinarian. The calving weight was 680 ± 62 (Mean ± STD) kg for MP cows and 550 ± 39 kg for PP cows. After calving, the PP and MP cows were separately assigned to three dietary groups, with the allocation in each age group balanced for predicted transmitting ability for fat plus protein (kg), pre-calving BW and BCS. The MP cows were also balanced for parity and previous lactation 305-day milk yield.

The cows were offered either (1) low concentrate (LC, 30% concentrate plus 70% grass silage, n = 6 for PP and n = 14 for MP); (2) medium concentrate (MC, 50% concentrate plus 50% grass silage, n = 6 for PP and n = 15 for MP) or (3) high concentrate (HC, 70% concentrate plus 30% grass silage, n = 6 for PP and n = 15 for MP) diets (percentages on a dry matter basis). The concentrate for each treatment was formulated to achieve a common total diet crude protein (CP) concentration for each of LC, MC and HC (152, 152 and 154 g/kg DM respectively), while the calculated total diet metabolisable energy (ME) contents were 12.0, 12.4 and 12.8 MJ/kg DM. The diets were estimated to supply 1556, 1997 and 2420 g/d effective rumen degradable protein; 559, 733 and 888 g/d dietary undegradable protein and 1346, 1817 and 2275 g/d metabolisable protein for LC, MC and HC, respectively. Access to treatment rations was controlled by a Calan Broadbent feeding system (American Calan Inc., Northwood, NH, USA) linked to an electronic identification system, which allowed individual cow intakes to be recorded daily. The diets for each treatment were offered at 107% of the previous days’ intake to ensure ad libitum consumption. All cows were also offered an additional 0.5 kg concentrate at each milking via an in-parlour feeding system to help maintain efficient cow flow. The present study was part of a wider project and full details of the diets offered, feeding management and feed composition were described previously [32].

### 4.2. Cow Phenotype Data Collection

Body weights were recorded twice weekly using weigh scales. BCS was estimated at around 14 DIM [100]. All cows were milked twice daily and their daily yields were recorded. Milk samples were analysed twice weekly using mid-infrared analysis for concentrations of protein, fat and lactose, and milk somatic cells were counted. Additional morning milk samples (2 × 8 mL) were collected twice weekly and stored at −18 °C for the analysis of LDH (EC. 1.1.1.27) and NAGase (EC 3.2.1.30) using fluorometric assays [101]. Energy-corrected milk yield (ECM; kg/day) was calculated following the methods used in our group [32]. The EBAL of each cow was estimated using the method described previously [102].

Clinical mastitis was diagnosed using standard methods based on daily observations for abnormal changes in milk appearance (e.g., flakes, clots), quality, milk yield and mammary inflammatory responses (redness, swelling, heat, or pain). Milk SCC readings together with the clinical diagnoses were used to categorise the cows into three groups. Healthy cows were defined as having an SCC < 100,000 cells/mL milk and no clinical symptom. Sub-clinically mastitic cows were defined as having an SCC between 100,000 and 400,000 cells/mL milk and no apparent clinical symptoms. Cows diagnosed as having clinical mastitis had an SCC > 400,000 cells/mL milk and showed some of the above clinical symptoms.

Cows were inseminated at observed estrus using normal herd practice and fertility data for the duration of the subsequent lactation or until the animal was culled were retrieved from the herd records. The data reported included days to first service (DFS), days to conception (days open), services per conception and the proportion of cows which conceived. In addition, the conception data were scored using a 4-point in calf by (ICB) score as (1) <100 days, (2) 100–200 days, (3) >200 days or (4) failed to conceive or culled.

### 4.3. Uterine Cytology Analysis

A uterine cytobrush sample (Minitube, Minitüb GmbH, Tiefenbach, Germany) was taken from every cow at around 14 DIM to evaluate endometrial cytology, as previously described [103]. A double guarded cytobrush was guided manually through the cervix into the uterus, the inner guard was extruded from the outer guard, and the brush was rotated gently against the endometrial wall. The brush was then withdrawn into the inner guard and removed. Slides for cytological examination were prepared by rolling the cytobrush onto a clean glass microscope slide and fixing the sample with Fisherbrand™ CytoPrep™ Cytology Fixative (Fishers Scientific, Blanchardstown, Ireland). Fixed slides were sent to the UCD School of Veterinary Medicine, University College Dublin, Ireland for processing and stained with modified Giemsa stain. Cytological assessment was performed by counting PMNs UECs at 400× magnification (Leitz Labourlux-S, Wetzlar, Germany) and determining their ratio, averaging counts of 10 high-power fields per slide. 

### 4.4. Analysis of Circulating Metabolites and IGF-1

At 14 ± 2 (mean ± STD) days after calving, 10 mL of blood samples were collected from the jugular vein of all cows into Na heparin tubes for plasma and plain tubes for serum. Following separation of plasma or serum with centrifugation, they were stored at −20 °C until analysis. Concentrations of plasma glucose, urea, BHB, NEFA and cholesterol were measured using the methods described previously [20,25]. Briefly, serum NEFA concentration was determined with the ACS-ACOD method using NEFA C Kits (Wako, Neuss, Germany). Plasma glucose was quantified with an enzymatic method (ADVIA 1800 Clinical Chemistry System, Siemens Healthcare Diagnostics, Ballerup, Denmark). Serum BHB was determined by measuring absorbance at 340 nm due to the production of NADH at alkaline pH in the presence of BHB dehydrogenase. Serum urea was analysed by spectrophotometry. Intra- and inter-assay coefficients of variation (CV) were in all cases below three and four percent, respectively, for both low and high control samples. Concentrations of serum IGF-1 were quantified with radioimmunoassay following acid-ethanol extraction [104]. Intra-assay CV were 12.4, 7.5 and 9.9% for low, medium and high control samples, respectively.

### 4.5. Blood RNA Extraction

Blood samples for RNA extraction were collected via jugular venepuncture from all cows at 14 ± 2 DIM into Tempus blood RNA tubes (Thermo-Fisher Scientific, Loughborough, UK). The tubes were shaken vigorously for 15–20 sec immediately upon collection, then frozen and stored at −80 °C for RNA extraction. The whole blood RNA was extracted using Tempus Spin RNA isolation Kits (Thermo-Fisher) following the manufacturer’s instruction as described previously [20]. An Agilent BioAnalyzer 2000 (Agilent Technologies UK Ltd., Cheadle, UK) with Agilent RNA 6000 Nano Kit (Agilent Technologies UK Ltd., Cheadle, UK) was used to assess the RNA quantity and integrity. In addition, the quantity and purity were validated with a NanoDrop 1000 (Thermo Fischer). Quality data are summarised in Supplementary File S3. This showed that all RNA samples had a reasonable integrity (RIN number > 8.7, 9.3 ± 0.3) and purity (260/280 between 2.01 and 2.15, mean ± STD 2.10 ± 0.03), so no animals were removed from the analysis. The RNA was stored at −80 °C for subsequent RNA-Sequencing.

### 4.6. RNA-Sequencing, Mapping and Quantification

The extracted leukocyte RNA was sequenced on the Illumina NextSeq 500 platform as described previously [68]. Briefly, using the epMotion liquid handling workstation (Eppendorf, Hamburg, Germany) 750 ng total RNA were reverse-transcribed into cDNA sequencing libraries with the Illumina TruSeq Stranded Total RNA Library Prep Ribo-Zero Gold kit (Illumina, San Diego, CA, USA). The pooled cDNA libraries were sequenced on an Illumina NextSeq 500 sequencer at 75 nucleotide length single end reads to reach an average of 33.5 million reads per sample. The raw FASTQ files were deposited to the European Nucleotide Archive (E-MTAB-9347 and E-MTAB-9431).

All sequencing analyses was carried out using CLC Genomic Workbench v21 (Qiagen, Manchester, UK). Each sample contained the reads from four lanes, and they were merged into one fastq file. The quality of both raw and trimmed fastq files were assessed following an Illumina Pipeline 1.8 and any failed reads were removed. Reads were then mapped to a reference genome Bos taurus assembly (ARS-UCD1.2 provided by RefSeq at https://www.ncbi.nlm.nih.gov/assembly, accessed on 1 January 2021) and quantified as reads per gene, reads per kilobase million (RPKM), and transcripts per kilobase million (TPM). These were stored as gene expression files (GE) in CLC Genomics Workbench to be used for the following analysis of differential gene expression.

### 4.7. Analysis of Differential Gene Expression between the Dietary Groups

Before the differential expression analysis for dietary effect, we used principal component analysis (PCA) with the RPKM values to identify the outliers, and this showed that two cows (Blood020009, MP and Blood020103, PP) were population outliers and were therefore excluded from further analysis (Supplementary File S4A). Principal component analysis also showed that there was only limited overlap in the overall gene expression pattern between the PP and MP cows (Supplementary File S2B). This supported the original study design to analyse each age group separately. The GE files derived from all individual samples were therefore separated by age group (PP, n = 5–6 per group and MP, n = 14–15 per group). The DEGs between the dietary groups in PP or MP cows were identified with CLC Genomics Workbench V21 via a one-way ANOVA-like procedure. False discovery rates (FDR) for multiple tests were adjusted with Benjamini–Hochberg (BH) and significance was considered at *p* < 0.05. The fold changes (FCs) were calculated as the gene expression ratio of higher concentrate group to lower concentrate group (e.g., HC vs. LC, MC vs. LC or HC vs. MC) if the value of the higher concentrate group was greater than that of the lower concentrate group (positive fold change, upregulation). If the value of lower concentrate group was greater than that of the higher concentrate group, the ratio of the lower concentrate group to the higher concentrate group was used (e.g., LC vs. HC, MC vs. HC and LC vs. MC) (negative fold change, downregulation). The DEGs upregulated and downregulated with an absolute fold change ≥ 1.5 between dietary groups were selected for further analysis.

### 4.8. Gene Ontology (GO) Enrichment Analysis

The DEGs derived from pair comparisons between the dietary groups were input into Partek Genomics Suite V7.1 (Partek Incorporation, Chesterfield, MO, USA) for GO enrichment analysis for investigation of the biological functions and interactions between the DEGs and the associated Kyoto Encyclopedia of Genes and Genomes (KEGG) pathways with a genome of Bos taurus ARS-UCD1.2. Fisher’s exact test with BH adjustment was used and statistical significance was considered at *p* < 0.05.

### 4.9. Statistical Analysis of Phenotype Data

The data derived from all cows were first split by the age group (PP and MP cows), according to the study design. The values of DMI, BW, milk parameters, EBAL, BCS, circulating metabolites (glucose, urea, BHB, NEFAs, cholesterol and IGF-1), SCC (logarithmic transformed) and uterine cell counts and their ratios were summarised as mean ± standard error of the mean (SE). Statistical analysis was used to compare their differences between the dietary groups using a one-way ANOVA built in SPSS V28 software package (Chicago, IL, USA). The homogeneity of variance for each variable was tested with Levene’s statistics before ANOVA. The results showed that homogeneity between groups for ECM, EBAL, glucose, urea, BHB and IGF-1 in the PP cows were not achieved, so logarithmic transformation was applied for these variables. Where ANOVA showed significance, multiple comparisons with Fisher’s LSD method was carried out to identify the source of differences. As homogeneity of the variance for the data of milk SCC and uterine cytology could not be achieved following logarithmic transformation, these variables were tested using a Kruskal–Wallis one way ANOVA with Dunn’s multiple comparison. The fertility data were tested with Wilcoxon method. In all cases, significance was considered at *p* < 0.05.

## 5. Conclusions

This study supports earlier work in demonstrating clear links between the metabolic status of cows in early lactation and their immune function. Most previous investigations which aimed at improving postpartum health through better nutrition have focused on the pre-partum period and demonstrated that overfeeding and high BCS at this time promote subsequent lipid mobilisation and an increased inflammatory response during the peripartum period [34]. We have instead assessed whether it is possible to alter immune functionality after calving by altering the lactation diet. We found that the response to additional concentrate inclusion, which provided a diet formulated to meet the animals energy and protein requirements, produced different effects on the leukocyte transcriptome in MP and PP cows. In the MP cows, the HC diet was clearly beneficial, as the leucocytes of the LC cow had upregulation of the complement and coagulation cascade and innate immune defence mechanisms against pathogens. In contrast, the leukocytes in the PP cows on the HC diet showed greater immune/inflammatory responses and they had a higher PMN:UEC ratio, suggesting increased leukocyte migration to the uterus. These cows subsequently had a longer interval of calving to conception, indicating poorer fertility. Further work with greater numbers of cows is warranted to confirm the finding of a dietary effect on fertility and to understand more fully how the metabolic responses of the postpartum diet differ in the younger animals.

## Figures and Tables

**Table 1:** Total dry matter intakes, milk parameters, body weights, energy balance, body condition score and blood metabolites at around 14 days after calving, analysed according to parity and diet ^1,2^.

	Multiparous			Primiparous		
Parameters ^3^	LC	MC	HC	LC	MC	HC
n	14	15	15	6	6	6
Total DMI (kg/d)	16.3 ± 0.8 ^c^	20.4 ± 0.9 ^b^	23.6 ± 0.9 ^a^	12.9 ± 0.6 ^b^	15.8 ± 0.52 ^a^	17.1 ± 0.6 ^a^
Body weight (kg)	656 ± 21	643 ± 14	669 ± 11	534 ± 16	536 ± 13	525 ± 17
Milk yield (kg/d)	33.4 ± 1.4 ^b^	35.9 ± 1.5 ^a,b^	37.9 ± 1.1 ^a^	20.7 ± 1.1	20.5 ± 2.0	23.3 ± 1.1
ECM (kg/d)	34.8 ± 1.6 ^b^	36.4 ± 1.5 ^a,b^	40.4 ± 1.4 ^a^	21.0 ± 1.2	22.0 ± 2.1	24.2 ± 1.2
EBAL (MJ/d)	−11.0 ± 1.5 ^b^	−4.6 ± 1.4 ^a^	−1.3 ± 1.3 ^a^	−4.5 ± 1.7 ^b^	0.1 ± 1.4 ^a,b^	1.6 ± 1.6 ^a^
BCS	2.64 ± 0.11	2.38 ± 0.08	2.53 ± 0.07	2.92 ± 0.11	2.88 ± 0.09	2.96 ± 0.15
Glucose (mmol/L)	3.30 ± 0.08 ^b^	3.34 ± 0.07 ^b^	3.61 ± 0.06 ^a^	3.82 ± 0.14	3.92 ± 0.10	3.98 ± 0.06
Urea (mmol/L)	3.47 ± 0.17 ^a^	3.01 ± 0.23 ^b^	2.54 ± 0.11 ^b^	4.38 ± 0.26 ^c^	2.70 ± 0.15 ^b^	2.17 ± 0.15 ^a^
BHB (mmol/L)	0.64 ± 0.08 ^a^	0.60 ± 0.06 ^a,b^	0.42 ± 0.05 ^b^	0.44 ± 0.03	0.40 ± 0.04	0.33 ± 0.04
NEFAs (mmol/L)	796 ± 104	656 ± 95	608 ± 81	502 ± 149	350 ± 99	462 ± 119
IGF-1 (ng/mL)	56 ± 7 ^b^	55 ± 7 ^b^	103 ± 10 ^a^	110 ± 22	160 ± 20	167 ± 29

^1^ Values are expressed as means ± SEM. a > b > c, *p* < 0.05–0.0001. ^2^ HC high concentrate, MC medium concentrate, LC low concentrate. ^3^ DMI dry matter intake, ECM energy-corrected milk yield, EBAL energy balance, BCS body condition score, BHB beta-hydroxybutyrate, NEFAs non-esterified fatty acids, IGF-1 insulin growth factor 1.

**Table 2:** Inflammatory parameters at around 14 days after calving according to parity and diet ^1,2^.

	Multiparous			Primiparous		
Parameters ^3^	LC	MC	HC	LC	MC	HC
n	14	15	15	6	6	6
Uterine PMNs/HPF	7.86 ± 1.85	8.82 ± 1.58	10.74 ± 2.11	4.68 ± 1.79	9.90 ± 3.16	10.03 ± 2.45
Uterine EC/HPF	12.49 ± 2.12	10.22 ± 2.02	9.09 ± 1.81	14.07 ± 1.48	7.25 ± 2.08	8.42 ± 2.31
Uterine PMNs:UEC	3.09 ± 1.49	3.56 ± 1.70	4.56 ± 1.76	0.39 ± 0.15 ^b^	1.90 ± 0.64 ^a,b^	2.10 ± 0.72 ^a^
Log_10_ SCC (1000 cells/mL)	1.85± 0.80 ^a^	1.55 ± 0.33 ^b^	1.32 ± 0.24 ^b^	1.74 ± 0.28	1.88 ± 0.51	2.09 ± 0.70
Cases of clinical mastitis ^4^ (n)	4	0	0	0	2	1
Cases of subclinical mastitis (n)	0	1	0	1	0	2
% clinical and subclinical mastitis	28.6%	6.7%	0%	16.7%	33.3%	50%
Milk Nagase (mU/mL)	2.99 ± 1.18	1.81 ± 0.12	1.78 ± 0.13	2.15 ± 0.18	2.40 ± 0.51	2.56 ± 0.46
Milk LDH (mU/mL)	5.20 ± 1.65	3.03 ± 0.28	2.93 ± 0.34	3.92 ± 0.44	4.79 ± 0.77	5.18 ± 0.88

^1^ Values are expressed as means ± SEM. a > b: *p* < 0.05–0.01. ^2^ HC high concentrate, MC medium concentrate, LC low concentrate. ^3^ PMNs: Polymorphonuclear leukocytes (neutrophils), EC: epithelial cells, SCC: somatic cell count, NAGase: N-acetyl-β-D-glucosaminidase, LDH: lactate dehydrogenase, HPF: high-power field. ^4^ Diagnosed within 16 days in milk.

**Table 3:** Fertility data according to parity and diet ^1,2^.

	Multiparous			Primiparous		
Parameters ^3^	LC	MC	HC	LC	MC	HC
n	14	15	15	6	6	6
DFS	80.4 ± 4.1	89.3 ± 6.7	91.1 ± 9.5	77.5 ± 6.4	76.8 ± 8.8	87.0 ± 8.4
Days open	117.0 ± 11.9	109.5 ± 8.0	108.7 ± 13.0	82.0 ± 5.1 ^b^	115.0 ± 22.7 ^a,b^	111.3 ± 1.3 ^a^
S/C	2.4 ± 0.4	1.8 ± 0.3	1.6 ± 0.2	1.0 ± 0.0	2.0 ± 0.5	2.0 ± 0.4
Not served (n)	2	3	4	0	0	2
FTC (n)	2	1	2	1	0	1
Pregnant (n)	10	11	9	5	6	3
ICB score ^4^	2.2 ± 0.3	2.1 ± 0.3	2.5 ± 0.4	1.5 ± 0.5 ^b^	1.3 ± 0.2 ^b^	3.0 ± 0.4 ^a^

^1^ Values are expressed as means ± SEM. a > b: *p* < 0.05. ^2^ HC high concentrate, MC medium concentrate, LC low concentrate. ^3^ DFS, days to first service; S/C, services per conception (of those which conceived); FTC, served but failed to conceive. ^4^ Conception data were scored using a 4-point in calf by (ICB) score as: (1) <100 days, (2) 100–200 days, (3) >200 days or (4) failed to conceive or culled.

**Table 4:** Summary of GO enrichment main functions of leukocyte DEGs in the comparison between the multiparous cows receiving high (n = 14) and low (n = 14) concentrate diets in early lactation.

Functions	Enrichment Score	Upregulated or Downregulated DEGs in the Function
Response to stimulus	10.9	Upregulated DEGs (12): *ALAS2*, *APLP1*, *COL1A1*, *COL1A2*, *COL3A1*, *GZMB*, *HTR1B*, *LIF*, *MS4A2*, *OR56A1*, *P2RY12*, *SPP1* Downregulated DEGs (33): *AHSG*, *ALB*, *ALOX5*, *APOA2*, *CELA2A*, *CFB*, *CYP1A2*, *FABP1*, *FGA*, *FGB*, *FGG*, *GATM*, *GLYAT*, *IFI6*, *IFNB1*, *ISG15*, *KNG1*, *MPO*, *MX2*, *NUPR1*, *OAS1Y*, *OAS1Z*, *OAS2*, *ORM1*, *PCK1*, *PRKCG*, *RBP4*, *RSAD2*, *SELENOM*, *TF*, *TRPC3*, *VTN*
Interspecies interaction between organisms	10.9	Upregulated DEGs (1): *GZMB* Downregulated DEGs (14): *APOA2*, *CFB*, *FGA*, *FGB*, *IFI6*, *IFNB1*, *ISG15*, *MPO*, *MX2*, *OAS1Y*, *OAS1Z*, *OAS2*, *PCK1*, *RSAD2*
Biomineralisation	7.1	Upregulated DEGs (4): *COL1A1*, *COL1A2*, *SPP1*, *TUFT1* Downregulated DEGs (0):
Immune system process	6.5	Upregulated DEGs (3): *GZMB*, *LIF*, *MS4A2* Downregulated DEGs (11): *ALOX5*, *CFB*, *FGA*, *FGB*, *IFI6*, *MPO*, *MX2*, *OAS1Y*, *OAS1Z*, *RSAD2*, *VTN*
Detoxification	5.9	Upregulated DEGs (1): *HBB* Downregulated DEGs (3): *FABP1*, *GSTA1*, *MPO*
Metabolic process	5.4	Upregulated DEGs (16): *ADAMDEC1*, *ADAMTS3*, *ALAS2*, *AOX1*, *APLP1*, *COL1A1*, *COL1A2*, *COL3A1*, *GZMB*, *IL1R2*, *LOC100139881*, *MAPK10*, *NRIP3*, *RNASE12*, *SPP1*, *ZNF215* Downregulated DEGs (31): *ACAN*, *ALDOB*, *ALOX5*, *APOA2*, *APOC3*, *APOH*, *CELA2A*, *CFB*, *CYP1A2*, *CYP2E1*, *FGB*, *FGG*, *GATM*, *GC*, *GLYAT*, *GSTA1*, *HMGCS2*, *HPX*, *IFI6*, *ISG15*, *ITIH2*, *MPO*, *NUPR1*, *PCK1*, *PPP1R3C*, *PRKCG*, *PROC*, *PTPN3*, *RBP4*, *SELENOM*, *TTR*
Multicellular organismal process	5.2	Upregulated DEGs (5): *ADAMTS3*, *COL1A1*, *COL3A1*, *HTR1B*, *SPP1* Downregulated DEGs (16): *AHSG*, *ALOX5*, *APOA2*, *APOC3*, *CLCN1*, *CYP1A2*, *FGB*, *FGG*, *GATM*, *KNG1*, *MPO*, *PRKCG*, *PROC*, *RBP4*, *SELENOM*, *SPP2*
Biological regulation	3.85	Upregulated DEGs (24): *ADAMTS3*, *APLP1*, *CDKN1C*, *COL1A1*, *COL1A2*, *COL3A1*, *DAB2*, *GCSAML*, *GZMB*, *HTR1B*, *IL1R2*, *IL20RA*, *LIF*, *LOC281376*, *MAPK10*, *MEIS3*, *MS4A2*, *MYCL*, *OR56A1*, *P2RY12*, *PDE9A*, *PPP1R1B*, *RSPO1*, *SPP1* Downregulated DEGs (43): *AHSG*, *ALB*, *ALDOB*, *ALOX5*, *AMBP*, *APOA2*, *APOC3*, *APOH*, *CELA2A*, *CFB*, *CLCN1*, *CYP1A2*, *DDX25*, *ELN*, *FABP1*, *FAM3B*, *FGB*, *FGG*, *GATM*, *HPX*, *IFI6*, *IFNB1*, *ISG15*, *ITIH2*, *KNG1*, *MX2*, *NUPR1*, *OAS2*, *OR12D2*, *ORM1*, *PCK1*, *PRKCG*, *PROC*, *PTPN3*, *RBP4*, *RHOBTB1*, *RSAD2*, *SELENOM*, *SERPINA3-7*, *TF*, *TRPC3*, *TTR*, *VTN*

**Table 5:** Significant pathways identified by KEGG pathway enrichment associated with differentially expressed leukocyte genes in the multiparous cows offered the high concentrate diet (n = 14) compared with those offered the low concentrate diet (n = 14).

Pathways	Enrichment FDR *p*-Value	Number of DEGs
Complement and coagulation cascades	9.972 × 10^−5^	8
Drug metabolism—cytochrome P450	1.853 × 10^−3^	5
Platelet activation	1.853 × 10^−3^	7
Protein digestion and absorption	4.322 × 10^−3^	6
ECM-receptor interaction	6.542 × 10^−3^	5
Metabolism of xenobiotics by cytochrome P450	1.200 × 10^−2^	4
Hepatitis C	1.200 × 10^−2^	6
Focal adhesion	1.207 × 10^−2^	7
Chemical carcinogenesis	1.377 × 10^−2^	4
Amoebiasis	1.377 × 10^−2^	5
Influenza A	1.377 × 10^−2^	7
Fc epsilon RI signalling pathway	2.039 × 10^−2^	4
PPAR signalling pathway	2.039 × 10^−2^	4
NOD-like receptor signalling pathway	2.433 × 10^−2^	6
Cholesterol metabolism	2.738 × 10^−2^	3
Glycolysis/Gluconeogenesis	4.341 × 10^−2^	3
AGE-RAGE signalling pathway in diabetic complications	4.341 × 10^−2^	4
Measles	4.341 × 10^−2^	5
Herpes simplex infection	4.341 × 10^−2^	6
Retinol metabolism	4.401 × 10^−2^	3
Thyroid hormone synthesis	4.401 × 10^−2^	3
Drug metabolism—other enzymes	4.942 × 10^−2^	3
Sphingolipid signalling pathway	4.942 × 10^−2^	4

**Table 6:** Summary of GO enrichment main functions of DEGs in the comparison between the primiparous cows offered the high (n = 6) and low (n = 6) concentrate diets in early lactation.

Functions	Enrichment Score	Upregulated and Downregulated DEGs in the Function
Immune system process	17.0	Upregulated DEGs (39): *ACOD1*, *ADAM8*, *ADGRG3*, *ARG2*, *BCL6*, *C1RL*, *C5AR1*, *C5AR2*, *CD14*, *CD24*, *CFP*, *CLEC4A*, *CLEC4D*, *CLEC4E*, *CXCL13*, *FES*, *HCK*, *HLX*, *HP*, *IL1A*, *LST1*, *LTF*, *MMP9*, *MSRB1*, *NDRG1*, *NFIL3*, *NLRP1*, *PGLYRP1*, *PGLYRP4*, *S100A12*, *S100A8*, *S100A9*, *SEMA4A*, *SKAP2*, *SLC11A1*, *THY1*, *TLR4*, *TREM1*, *TYROBP* Downregulated DEGs (5): *BCAR1*, *CDH17*, *NKG7*, *PRF1*, *SRMS*
Locomotion	15.2	Upregulated DEGs (16): *ADAM8*, *C5AR1*, *C5AR2*, *CCL16*, *CXCL13*, *CXCR1*, *CXCR2*, *DEFB1*, *DEFB10*, *DEFB7*, *NRG1*, *NRP1*, *PROK2*, *PTAFR*, *S100A8*, *S100A9* Downregulated DEGs (2): *BCAR1*, *WNT5A*
Interspecies interaction between organisms	10.3	Upregulated DEGs (27): *ARG2*, *C5AR1*, *CATHL6*, *CD14*, *CFP*, *CLEC4D*, *CLEC4E*, *CLEC5A*, *CXCL13*, *DEFB1*, *FN1*, *HMOX1*, *HP*, *LTF*, *MSRB1*, *NECTIN2*, *NLRP1*, *NRP1*, *PGLYRP1*, *PGLYRP4*, *S100A12*, *S100A8*, *S100A9*, *SCARB1*, *SLC11A1*, *TLR4*, *TREM1* Downregulated DEGs (3): *GZMA*, *NKG7*, *PRF1*
Cellular process	5.7	Upregulated DEGs (210): *ABCA6*, *ACSL6*, *ACVR1B*, *ADAM8*, *ADCY6*, *ADGRG3*, *ALDH1L2*, *ALOX5AP*, *ALPK1*, *ALPK3*, *AMPD3*, *ANGPTL3*, *ANKS4B*, *APH1B*, *ARAP3*, *ARG2*, *ARHGAP22*, *ARRDC4*, *ASS1*, *ATF3*, *BCL6*, *BMX*, *C5AR1*, *C5AR2*, *CAMKK1*, *CAPN3*, *CARMIL1*, *CATHL6*, *CCR1*, *CD14*, *CD24*, *CDS1*, *CHI3L1*, *CLEC4A*, *CLEC4D*, *CLEC4E*, *CLEC5A*, *COL1A1*, *COL1A2*, *COL3A1*, *CREB3L2*, *CRISPLD2*, *CTBP2*, *CXCL13*, *CXCR1*, *CXCR2*, *DACH1*, *DAGLB*, *DCK*, *DCN*, *DEFB1*, *DGAT2*, *DGKG*, *DIRAS3*, *DISC1*, *DLC1*, *DNER*, *DOCK4*, *DPYD*, *DUSP1*, *DYSF*, *EGR2*, *EIF4E3*, *EIF4EBP1*, *ELN*, *ENKUR*, *EREG*, *ETV5*, *FAT1*, *FES*, *FLT3*, *FMO2*, *FN1*, *FOS*, *FOSL2*, *GAB2*, *GADD45A*, *GGT5*, *GK*, *GPAT3*, *GPR27*, *GPR87*, *GPT2*, *GPX3*, *GSTO2*, *HAL*, *HCK*, *HEPACAM2*, *HK3*, *HLX*, *HMOX1*, *HP*, *HRH2*, *ICAM3*, *IFIT3*, *IL17RD*, *IL1A*, *IL1RAP*, *IL1RN*, *ISG20*, *KCNJ2*, *KREMEN1*, *LAMA3*, *LGR6*, *LOC100337213*, *LOC112447333*, *LOC514257*, *LOC521224*, *LOC527744*, *LOC538435*, *LTF*, *MAP3K6*, *MAPK13*, *MARCKSL1*, *MMP8*, *MMP9*, *MPV17L*, *MSRB1*, *MZB1*, *NAIP*, *NDRG1*, *NECTIN2*, *NFAM1*, *NFIL3*, *NLRP1*, *NRP1*, *OR52B2*, *OR52L1*, *OR52W1*, *OSCAR*, *PADI4*, *PCSK1*, *PDXK*, *PDZD3*, *PFKFB4*, *PGLYRP1*, *PGLYRP4*, *PIGR*, *PKD2*, *PLAUR*, *PLB1*, *PLIN3*, *PLXND1*, *PPP1R3B*, *PRKAA2*, *PRODH*, *PROK2*, *PTAFR*, *PTPN5*, *RAB20*, *RAB3D*, *RAB3IP*, *RBM47*, *RECK*, *RETN*, *RIDA*, *RND3*, *RPH3A*, *S100A12*, *S100A8*, *S100A9*, *SCARB1*, *SDS*, *SEMA4A*, *SEMA7A*, *SERINC2*, *SERPINE1*, *SH3PXD2B*, *SKAP2*, *SLC11A1*, *SLC13A3*, *SLC13A5*, *SLC16A3*, *SLC16A5*, *SLC28A3*, *SLC2A9*, *SLC30A4*, *SLC40A1*, *SLC5A9*, *SLC6A9*, *SLC8A1*, *SLCO4C1*, *SNX18*, *SNX24*, *SOCS3*, *SOCS6*, *SORT1*, *SPATA6*, *SRGN*, *STARD9*, *STEAP4*, *TARM1*, *TBXAS1*, *TCN1*, *TGFA*, *TGFBI*, *THY1*, *TJP2*, *TLR4*, *TNFAIP6*, *TNFRSF8*, *TYROBP*, *UGGT2*, *UPP1*, *USP35*, *VCAN*, *VLDLR*, *WDFY3*, *WIPI1*, *WLS* Downregulated DEGs (28): *ADGRG1*, *AOX1*, *ATP6V0A4*, *BACE1*, *BCAR1*, *CA8*, *CCR5*, *CD96*, *CDH17*, *CHN1*, *EPCAM*, *GRM8*, *GSDMA*, *GZMA*, *LOC784535*, *NKG7*, *PRF1*, *S1PR5*, *SAXO1*, *SCN11A*, *SLC22A23*, *SLC38A11*, *SLC6A15*, *SLC7A4*, *SRMS*, *SRPK3*, *TDRKH*, *WNT5A*, *ZWILCH*
Detoxification	4.5	Upregulated DEGs (6): *ALOX5AP*, *GPX3*, *GSTO2*, *HP*, *S100A8*, *S100A9* Downregulated DEGs (0)
Biological regulation	3.5	Upregulated DEGs (115): *A2M*, *ACOD1*, *ACVR1B*, *ADAM8*, *ADGRG3*, *ALPK1*, *ANGPTL3*, *APH1B*, *ARG2*, *ARRDC4*, *ATF3*, *BASP1*, *BCL6*, *BST1*, *C1RL*, *C5AR1*, *C5AR2*, *CAPN3*, *CARMIL1*, *CCR1*, *CD101*, *CD14*, *CD24*, *CFP*, *CHI3L1*, *CLEC4A*, *CLEC4D*, *CLEC4E*, *CLEC5A*, *COL1A1*, *COL3A1*, *CREB3L2*, *CREB5*, *CTBP2*, *CXCL13*, *DACH1*, *DAGLB*, *DCN*, *DEFB1*, *DGKG*, *DISC1*, *DLC1*, *DOCK4*, *DUSP1*, *EGR2*, *EIF4EBP1*, *EREG*, *ETV5*, *FES*, *FMO2*, *FN1*, *FOS*, *FOSL2*, *GADD45A*, *GPAT3*, *GPR27*, *HCK*, *HLX*, *IL17RD*, *IL1A*, *IL1RAP*, *KREMEN1*, *LAMA3*, *LGR6*, *LOC100298356*, *LOC101903018*, *LOC515676*, *LRP1*, *LTF*, *MAPK13*, *MMP8*, *MMP9*, *MN1*, *MPV17L*, *MXD1*, *MZB1*, *NAIP*, *NECTIN2*, *NFAM1*, *NFIL3*, *NLRP1*, *NLRP12*, *NRG1*, *NRP1*, *PDZD3*, *PIGR*, *PLAUR*, *PPP1R3B*, *PRKAA2*, *RAP1GAP*, *RECK*, *RETN*, *RIDA*, *S100A8*, *S100A9*, *SCARB1*, *SEMA4A*, *SEMA7A*, *SLC11A1*, *SNX18*, *SOCS3*, *SOCS6*, *SORT1*, *TARM1*, *TGFA*, *THY1*, *TLR4*, *TNFAIP6*, *TNFRSF8*, *TYROBP*, *VLDLR*, *WIPI1*, *WLS*, *WWC2*, *ZNF114* Downregulated DEGs (10): *ADGRG1*, *BCAR1*, *CD96*, *CHN1*, *EOMES*, *EPCAM*, *FCRL3*, *GZMA*, *NKG7*, *WNT5A*
Signalling	3.2	Upregulated DEGs (6): *CXCL13*, *EREG*, *IL1RAP*, *KCNJ2*, *PKD2*, *THY1* Downregulated DEGs (2): *CCR5*, *WNT5A*

**Table 7:** Significant pathways identified by Kegg pathway enrichment associated with differentially expressed leukocyte genes in the primiparous cows offered the high concentrate diet (n = 6) compared with those offered the low concentrate diet (n = 6).

Pathways	Enrichment FDR *p*-Value	Number of DEGs
Osteoclast differentiation	1.660 × 10^−4^	11
Relaxin signalling pathway	3.384 × 10^−4^	10
Amoebiasis	4.880 × 10^−4^	9
Fluid shear stress and atherosclerosis	1.435 × 10^−3^	10
Valine, leucine and isoleucine biosynthesis	1.508 × 10^−3^	2
Chemokine signalling pathway	5.046 × 10^−3^	10
ECM-receptor interaction	5.677 × 10^−3^	6
AGE-RAGE signalling pathway in diabetic complications	8.527 × 10^−3^	7
Vitamin B6 metabolism	8.580 × 10^−3^	2
Growth hormone synthesis, secretion and action	9.80 × 10^−3^	7
Arginine biosynthesis	1.096 × 10^−2^	3
Biosynthesis of amino acids	1.124 × 10^−2^	5
Platelet activation	1.129 × 10^−2^	7
Aldosterone synthesis and secretion	1.326 × 10^−2^	6
Cortisol synthesis and secretion	1.355 × 10^−2^	5
Drug metabolism—other enzymes	1.549 × 10^−2^	5
Cholesterol metabolism	1.743 × 10^−2^	4
Cholinergic synapse	1.959 × 10^−2^	6
Protein digestion and absorption	2.162 × 10^−2^	6
Glutathione metabolism	2.364 × 10^−2^	4
MAPK signalling pathway	2.376 × 10^−2^	12
Drug metabolism—cytochrome P450	2.479 × 10^−2^	4
Arachidonic acid metabolism	2.504 × 10^−2^	5
TNF signalling pathway	3.116 × 10^−2^	6
PI3K-Akt signalling pathway	3.183 × 10^−2^	14
Salmonella infection	3.656 × 10^−2^	5
Pertussis	3.656 × 10^−2^	5
Longevity regulating pathway	3.656 × 10^−2^	5
Amphetamine addiction	4.122 × 10^−2^	4
Glycerolipid metabolism	4.282 × 10^−2^	4
Dopaminergic synapse	4.310 × 10^−2^	6
Phospholipase D signalling pathway	4.439 × 10^−2^	7
Complement and coagulation cascades	4.534 × 10^−2^	5
Thyroid hormone synthesis	4.610 × 10^−2^	4
Pantothenate and CoA biosynthesis	4.805 × 10^−2^	2

## Data Availability

The RNA-seq fastq data can be obtained from the European Nucleotide Archive at https://www.ebi.ac.uk/ena/browser/home (accessed on 1 May 2022) with the accession number of E-MTAB-9347 and E-MTAB-9431.

## Supplementary Materials

The following supporting information can be downloaded at: https://www.mdpi.com/article/10.3390/ijms24010039/s1. Supplementary File S1A–E (A: Differential expressed genes between the MP cows offered high and low concentrate, B: Differential expressed genes between the MP cows offered medium and low concentrate, C: Differential expressed genes between the MP cows offered high and medium concentrate, D: Top 20 upregulated circulating leukocyte genes in the MP cows fed with high concentrate compared with those fed with low concentrate as ranked by the *p* values, E: Top 20 downregulated circulating leukocyte genes in the multiparous cows fed with high concentrate compared with those fed with low concentrate as ranked by the *p* values); Supplementary File S2A–F (A: Differentially expressed leukocyte genes between the primiparous cows offered high and low concentrate diets, B: Differentially expressed leukocyte genes between the primiparous cows offered medium and low concentrate diets, C: Differentially expressed leukocyte genes between the primiparous cows offered high and medium concentrate diets, D: Top 20 upregulated circulating leukocyte genes in the primiparous cows fed with high concentrate compared with those fed with low concentrate as ranked by the *p* values, E: Top 20 downregulated circulating leukocyte genes in the primiparous cows fed with high concentrate compared with those fed with low concentrate as ranked by the *p* values), F: Upregulated DEGs between the primiparous cows offered high (n = 6) and low (n = 6) concentrate diets involved in top 20 GO functions); Supplementary File S3: RNA quality; Supplementary File S4: Principal component analysis.
