# Supplementary material for: Proportion of Concentrate in the Diet of Early Lactation Dairy Cows Has Contrasting Effects on Circulating Leukocyte Global Transcriptomic Profiles, Health and Fertility According to Parity
Source: Int J Mol Sci. 2022 Dec 20;24(1):39. doi: 10.3390/ijms24010039 (PMC9820068; doi:10.3390/ijms24010039)
Supplement: Supplementary file 1 [file ijms-24-00039-s001.zip › Supplementary File S4.pdf]

A

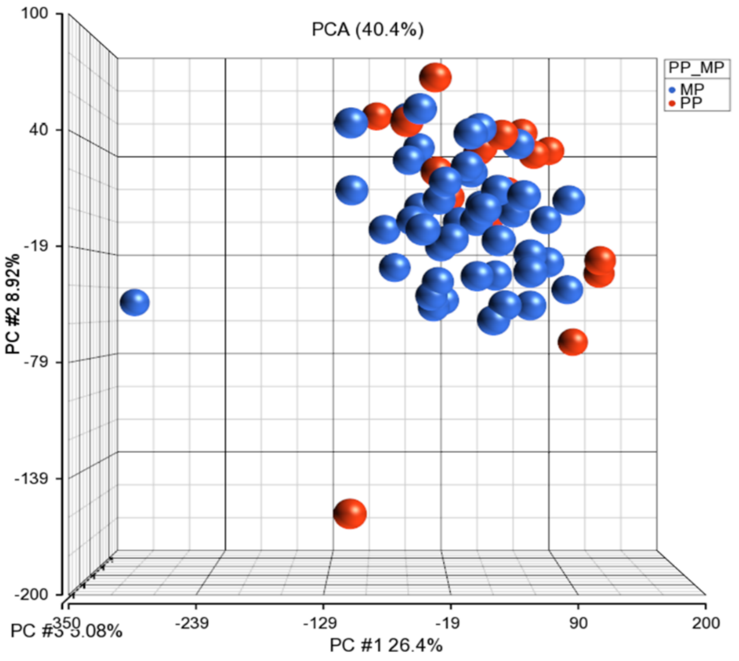

B

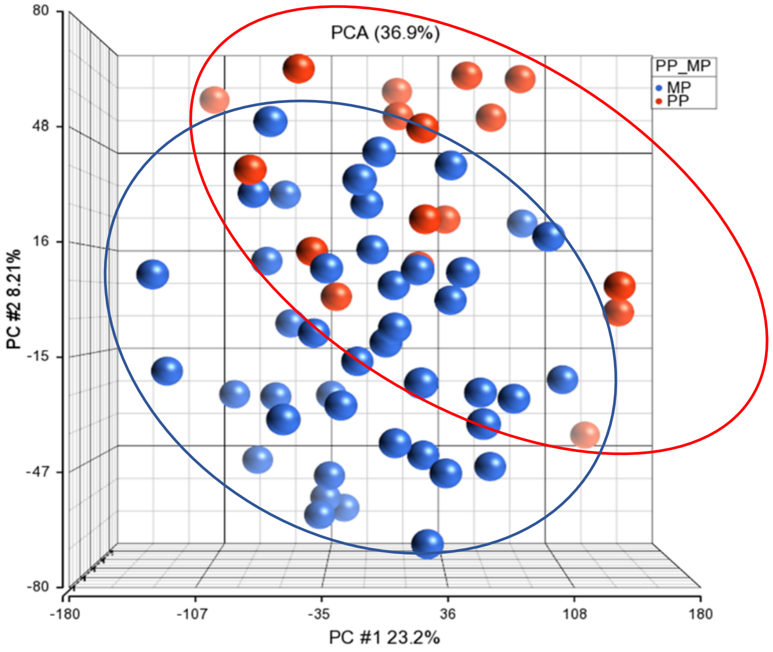

Supplementary File S4. Principal component analysis with the normalized read counts (RPKM) showing (A) the outliers for one primiparous (PP) and one multiparous (MP) cows and (B) the differences of overall leukocyte gene expression between PP and MP cows after removing of the outliers.
